# ARTS-DB: a database for antibiotic resistant targets

**DOI:** 10.1093/nar/gkab940

**Published:** 2021-10-28

**Authors:** Mehmet Direnç Mungan, Kai Blin, Nadine Ziemert

**Affiliations:** Interfaculty Institute of Microbiology and Infection Medicine, Institute for Bioinformatics and Medical Informatics, University of Tübingen, 72076 Tübingen, Germany; German Center for Infection Research (DZIF), Partner Site Tübingen, 72076 Tübingen, Germany; The Novo Nordisk Foundation Center for Biosustainability, Technical University of Denmark, Kemitorvet Bygning 220, 2800 Kgs. Lyngby, Denmark; Interfaculty Institute of Microbiology and Infection Medicine, Institute for Bioinformatics and Medical Informatics, University of Tübingen, 72076 Tübingen, Germany; German Center for Infection Research (DZIF), Partner Site Tübingen, 72076 Tübingen, Germany

## Abstract

As a result of the continuous evolution of drug resistant bacteria, new antibiotics are urgently needed. Encoded by biosynthetic gene clusters (BGCs), antibiotic compounds are mostly produced by bacteria. With the exponential increase in the number of publicly available, sequenced genomes and the advancements of BGC prediction tools, genome mining algorithms have uncovered millions of uncharacterized BGCs for further evaluation. Since compound identification and characterization remain bottlenecks, a major challenge is prioritizing promising BGCs. Recently, researchers adopted self-resistance based strategies allowing them to predict the biological activities of natural products encoded by uncharacterized BGCs. Since 2017, the Antibiotic Resistant Target Seeker (ARTS) facilitated this so-called target-directed genome mining (TDGM) approach for the prioritization of BGCs encoding potentially novel antibiotics. Here, we present the ARTS database, available at https://arts-db.ziemertlab.com/. The ARTS database provides pre-computed ARTS results for >70,000 genomes and metagenome assembled genomes in total. Advanced search queries allow users to rapidly explore the fundamental criteria of TDGM such as BGC proximity, duplication and horizontal gene transfers of essential housekeeping genes. Furthermore, the ARTS database provides results interconnected throughout the bacterial kingdom as well as links to known databases in natural product research.

## INTRODUCTION

Throughout history, humanity has been in a constant battle with bacteria causing infectious diseases (1). Especially in the last decades, due to the escalation of multi-drug resistant bacteria, these continuously evolving pathogens have become a serious threat to human health. Consequently, there is an urgent need for novel antibiotics with new modes of action (2,3). Secondary metabolites (SMs) are the key molecules feeding antimicrobial drug development pipelines (4). These so-called natural products, are profusely found and isolated from fungal and bacterial organisms (5). The discovery of natural products has traditionally been centered on bioactivity screening. With the advent of genome sequencing in the last decade or two, *in silico* methods can now be used to complement these approaches. Presently, genome mining offers a wide range of computational applications that predict the biosynthetic gene clusters (BGCs) encoding enzymes necessary for the formation of natural products (6,7). Adopting algorithmic architectures like deep learning and hidden markov models, BGC prediction tools such as antiSMASH (8), PRISM (9) or DeepBGC (10), have been used in natural product research for over a decade. As a result of the genome mining efforts, hundreds of thousands of BGCs are continuously deposited in publicly available databases such as antiSMASH-DB (8) and Atlas of Biosynthetic Gene Clusters (IMG-ABC). The total of experimentally verified genome-mined BGCs however, falls even below 1% (11). Since experimental validation of a BGC and its compound is a labour-intensive process (12), a crucial task now is the prioritization of BGCs for further downstream analysis.

A recently established technique adopts a BGC prioritization approach leveraging the idea that in order to avoid suicide, bacteria need to be evolved in such a way that they are resistant to the compounds they produce (13). One of the resistance mechanisms bacteria use to protect themselves from their own bioactive compounds is the modification of the antibiotics target (14). In such processes, the duplicated and modified antibiotic target gene can be found within the BGC, providing self resistance (15,16). This so-called target-directed genome mining (TDGM) approach allowed researchers to predict the mode of action of the compounds encoded by uncharacterized BGCs and led to the identification of new natural products (17–19). Since 2017, the Antibiotic Resistant Target Seeker (ARTS) facilitated TDGM approaches in order to prioritize promising strains producing antibiotics with putative novel modes of action by rapidly linking housekeeping and known resistance genes to BGC proximity, duplication and horizontal gene transfer (HGT) events (20,21). By design, the ARTS pipeline functions as a web-server, analyzing user supplied genomes individually with a ‘one job at a time’ mentality which takes a certain processing time. In order to further improve our work on self resistance genome mining, we have developed the ARTS database, a user-friendly web-server for the extensive exploration of the bacterial kingdom using TDGM approaches. The ARTS database provides a global picture of ARTS results interconnected with the whole kingdom of bacteria and provides connections between potential targets and relevant databases containing additional information about respective BGCs or existing drugs. Currently, the ARTS database contains pre-computed ARTS results for a total of 27,096 high quality bacterial genomes obtained from NCBI’s RefSeq database (22), also present in the antiSMASH-DB. Given that there is an ever-increasing usage of metagenomic applications on natural product research, we have also included 43,130 metagenome assembled genomes (MAGs) in the ARTS database described by Nayfach *et al.* (23).

The ARTS database allows researchers to facilitate TDGM based exploration through two main search functions. One of them is the exploration of fundamental ARTS hits such as BGC proximity, duplication and HGT evidence by using a query builder. All of the returned sequences are linked to individual ARTS and antiSMASH results for closer inspection. Second, a target-oriented exploration can be made. Here, the user can search a gene of interest throughout the database, in order to find phylogenetical and statistical information about a potential resistant target with respect to bacterial kingdom.

## DATABASE DESIGN

Using a multi-layered setup, the ARTS database provides rapid execution of provided queries using SQLAlchemy toolkit (https://www.sqlalchemy.org/) for relational mapping on a Flask-based framework (https://flask.palletsprojects.com/). The whole database is originally stored using SQLite database engine (https://www.sqlite.org/). The front end is comprised of jquery, bootstrap and ajax for high compatibility between different devices and browsers. The web service layer allows for easy execution of SQL logic packed in a single page. All ARTS results can be linked via web application and are stored on a disk hosted by de.NBI cloud (24).

### Genomic sequence content

**Figure 1.**
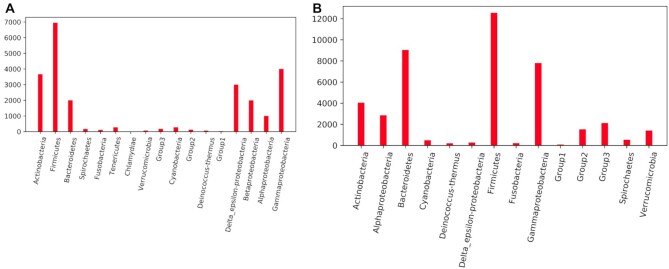
Included genome counts by reference set. Panels (**A**) and (**B**) show the phylogenetic distribution of genomes acquired from data sources NCBI RefSeq and GEM, respectively. Genome contents of the reference sets termed as ‘Group’ comprise underexplored phyla of the bacterial kingdom and described in detail in latest ARTS publication (21).

The ARTS database includes genomic sequences, fueled by two different repositories. One of them is NCBI’s publicly available RefSeq database (22) whose bacterial genomes are also used by the antiSMASH-DB. Selection and filtering of the genomes are explained in detail in the latest version of the antiSMASH-DB described by Blin *et al.* (8). In summary, the ARTS database contains 27,096 high quality bacterial genomes (Figure 1A) which were selected according to their completeness level. To discard fragmented and low quality assemblies, genomes labeled as complete assembly or with contig count <100 were included in the database. Using MASH (25), redundant sequences were also filtered out with a similarity cutoff of 99.6%.

Additionally, the ARTS database covers sequences from metagenomes. In a recent study published in 2021, Nayfach and his colleagues explored microbiomes from a wide range of habitats all around the Earth as well as mammalian hosts, forming the Genomes from the Earth’s Microbiomes (GEM) catalogue. GEM has supplied the community with >52,000 MAGs and their genome mining data regarding BGCs deposited in IMG/M (26), greatly increasing the existing knowledge about secondary metabolite biosynthetic potential of microorganisms. However, for an accurate housekeeping gene search, ARTS pipeline is dependent on reference sets which were built using closely related taxa. Therefore, it doesn’t guarantee high accuracy for bacteria that are assigned to a candidate phylum. For ARTS database, we have selected >43,000 MAGs based on their taxonomic annotation via GTDB (27) (Figure 1B) that fit the ARTS reference sets.

## MAIN APPLICATIONS

**Figure 2.**
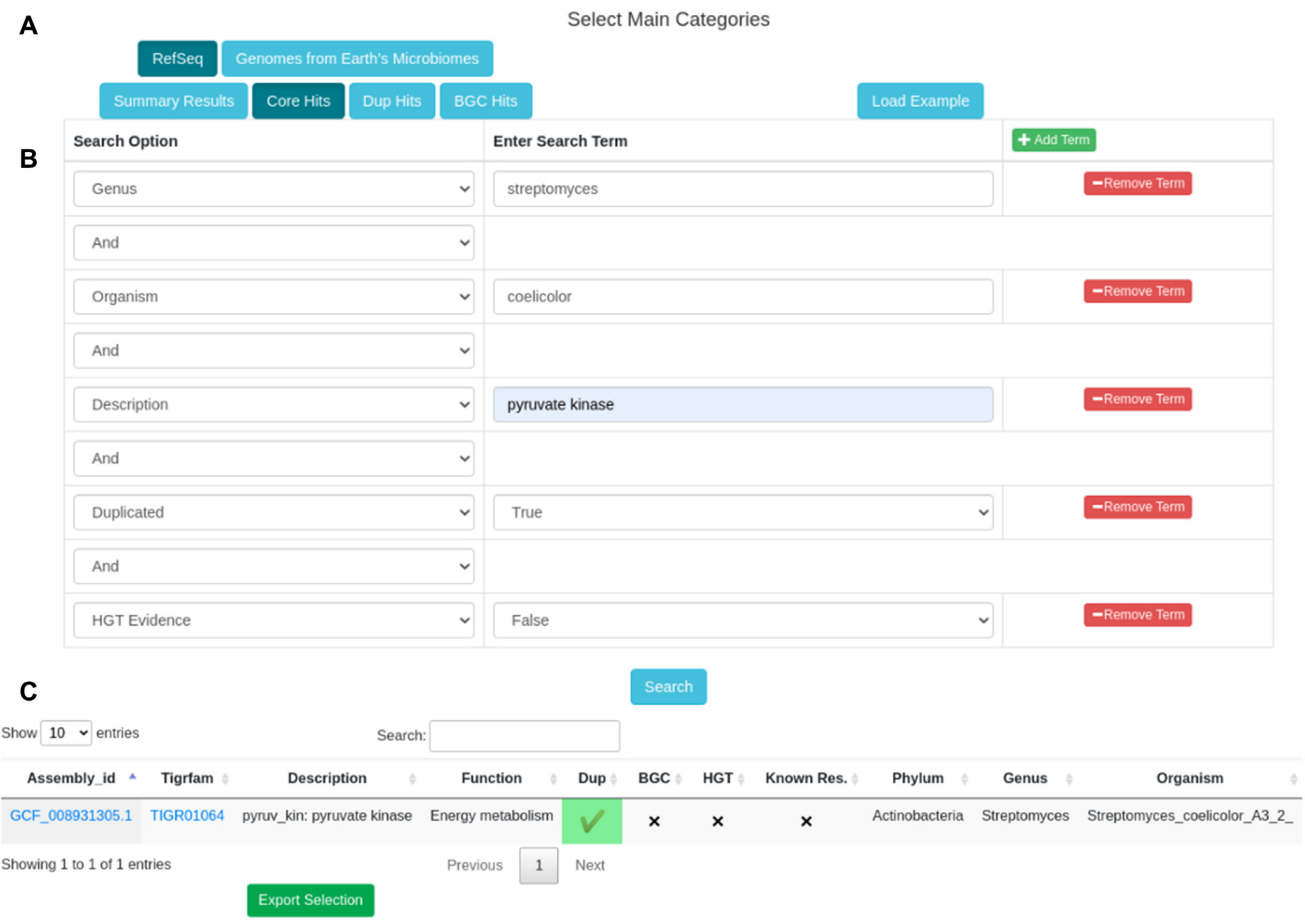
Query example in the ARTS database. (**A**) One of the available data sources ‘RefSeq’ and ‘Genomes from Earth’s Microbiomes’ and one of the four main routes below to explore selected data source must be selected. (**B**) After selecting main categories, search options and terms must be specified by using the ‘Add Term’ button. (**C**) The example output.

As mentioned earlier, the ARTS database offers two search options: ‘Query Building’ and ‘Target-Oriented Search’. Using a query builder, users can explore available data sources in the ARTS database through four main routes (Figure 2A). These routes allow for: generating statistical summaries of ARTS results for the initial filtering of genomes of interest, finding essential housekeeping genes that have hits for fundamental ARTS criteria, exploring duplication rates of a gene of interest based on its occurrence frequency in different phyla as well as an essential genes function and frequency in different BGCs. Complex queries can be easily built by using the ‘Add Term’ button and adding the conditions indicating advanced properties of the search. The resulting tables can also be filtered, sorted or searched dynamically, allowing easy navigation through the resulting potential targets.

In addition, the ‘Target-Oriented Search’ option gives a broader view about the characteristics of the selected gene such as its proximity to different BGC types or in which phyla it is considered as an essential housekeeping gene. In order to maintain a high level of inter-operability, the ARTS database offers cross-links to available repositories such as MIBiG (28) and BiGFAM (29) for exterior information about a predicted BGC and its cluster families, respectively. Furthermore, DrugBank (30) entries are provided where applicable, for additional information about a genes affiliation with existing drugs and their known modes of action.

### Building queries

#### Case study

In a recent study, Hoskisson *et al.* investigated how the expansion of primary metabolism plays a role in the biosynthesis of antibiotics (31). In order to find gene expansion events in primary metabolism pathways, they analyzed 612 actinobacterial genomes to generate gene frequencies for 60 genera. Of note, they were exclusively interested to gene expansions through duplication but not via HGT. After going through extensive bioinformatic pipeline sessions to satisfy such requisites, their analysis pointed them towards a duplicated pyruvate kinase in *Streptomyces coelicolor* A3(2), for further evaluation. Using the ARTS database, such enquiries can be made in seconds.

#### Duplication search

In order to execute such a query, after selecting data source as ‘RefSeq’ and search category as ‘Dup Hits’, the user can click on the ‘Add Term’ button to start shaping the search. For example, after adding ‘Genus’ search option with the term ‘streptomyces’ and pressing ‘Search’, the user will have access to duplication rates of all essential genes from the genus streptomyces, including the gene counts in specific organisms, average gene counts for the reference set and its standard deviation. Afterward, dynamic filtering of the results for specific organisms or genes can easily be done by simply typing ‘coelicolor pyruvate kinase’ in the ‘Search’ box. However, it is advised to shape the initial search with parameters of interest since it will ease the browser’s memory usage down and increase the execution speed of the query.

#### Core hit attributes

After detecting the gene of interest that shows statistical evidence for the duplication event, the user can easily check whether the gene fits in with other aspects of TDGM, here, an HGT event (Figure 2B). In our case, such query can be made using ‘Core Hits’ tab this time with the ‘Genus’ option with the term ‘streptomyces’ and adding the ‘Description’ option with the term ‘pyruvate kinase’ and simply adding the search option ‘HGT Evidence’ set to ‘False’. Resulting table will only contain the gene of interest with direct links to individual ARTS result of the genome and HMM model of the gene for closer inspection (Figure 2C).

#### Further examination

The ARTS database provides opportunities for closer inspection of the resulting queries. For example, if the user is interested in BGCs that contain the gene of interest, the ‘BGC Hits’ tab can be used with the same search options to retrieve BGC specific results. Thereafter, the user can check the antiSMASH results of specific clusters, their gene cluster families in BiGFAM database consisting of closely related BGCs or the complete ARTS result, using the provided links. Items in the column ‘Model Name’ will lead to the target-oriented result page. Here, the user can explore the characteristics of a specific target gene and its fundamental ARTS criteria hits, with respect to the phyla where it is considered as an essential housekeeping gene. Moreover, commercially available drugs targeting the genes of interest are also shown through the links connected to the DrugBank database as well as the known BGCs that contain the gene via links to the MIBiG database. All of the resulting tables and individual ARTS results can be downloaded in order to feed in-house analysis pipelines.

## CONCLUSIONS AND FUTURE PERSPECTIVES

With the continuous advancements in genome sequencing techniques and BGC prediction algorithms, genome mining applications have become a vital factor in natural product research. A recently developed self resistance based approach, is progressively used by researchers for the discovery of natural products with novel modes of action. Since its first release in 2017, ARTS has been allowing researchers to rapidly mine their sequences with self resistance based genome mining approaches. Currently, to the best of our knowledge, ARTS is the only webserver enabling such method in all bacteria. Here, we present the ARTS database, a comprehensive repository containing a high quality bacterial genome set from NCBI’s RefSeq and GEM catalogue processed with TDGM strategies. The ARTS database now allows researchers to quickly access pre-computed ARTS results and explore the bacterial kingdom via a broader view.

For future work, in order to further improve ARTS and the ARTS database, we are in the process of making ARTS analysis available for fungal genomes as well. We are also developing complementary tools such as SYN-view (32) for the enhancement of the ARTS pipeline and increasing its accuracy using additional criteria. Since the need for new antibiotics and the usage of genome mining methodologies increase on a daily base, we are confident that the ARTS database will be a resource of significant importance in the search for novel natural products.

## DATA AVAILABILITY

The ARTS database is publicly available online at https://arts-db.ziemertlab.com/ with no access restrictions. All of the source code involving Python and JS scripts as well as HTML content is available on Bitbucket at https://bitbucket.org/mehmetdirenc/arts_database/. All the accessions and queries are safely executed via HTTPS protocol.
